# The complete chloroplast genome sequence of *Begonia coptidifolia*

**DOI:** 10.1080/23802359.2021.1872434

**Published:** 2021-02-12

**Authors:** Zheng-Feng Wang, Ting-Hong Liu, Hong-Lin Cao

**Affiliations:** a Key Laboratory of Vegetation Restoration and Management of Degraded Ecosystems, South China Botanical Garden, Chinese Academy of Sciences, Guangzhou, China; b Center for Plant Ecology, Core Botanical Gardens, Chinese Academy of Sciences, Guangzhou, China; c Southern Marine Science and Engineering Guangdong Laboratory (Guangzhou), Guangzhou, China; d University of Chinese Academy of Sciences, Beijing, China

**Keywords:** *Begonia coptidifolia*, chloroplast, genome assembly, high-throughput sequencing

## Abstract

*Begonia* (Begoniaceae) is a large, pantropically distributed genus, comprising more than 1900 species. Due to poorly available genome resources, the phylogeny of this species-rich genus is still challenged. *B*. *coptidifolia* is a newly discovered species of restricted distribution in Southern China, and its genetic relationship with the other *Begonia* species has not been reported. Therefore, in this study, we report for the first time its chloroplast genome for future phylogenetic analysis. The circular chloroplast genome of *B*. *coptidifolia* is 169,412 bp in length, with a GC content of 35.57%. Its large single-copy region is 75,937 bp, a small single-copy region is 18,362 bp, and two inverted repeat regions are 37,556 bp and 37,557 bp, respectively. The genome encodes 82 protein-coding genes, 8 ribosomal RNA genes, and 40 transfer RNA genes. Phylogenetic analysis indicated that *B*. *coptidifolia* is genetically closest to *B*. *pulchrifolia*.

The genus *Begonia* (Begoniaceae) is one of the 10 largest and fastest-growing genera (Ye et al. 2004; Moonlight et al. 2020). It presently includes more than 1900 species (Moonlight et al. 2020), of which approximately 370 are distributed in China. The species of *Begonia* are herbs, shrubs, and epiphytes, and they have a widespread distribution in tropical and subtropical areas. Many *Begonia* species are important ornamental plants. Some of them have asymmetrical, patterned, and variegated foliage, and others are attractive with their bright blooms. Despite many molecular evolutionary analyses using different genetic markers on *Begonia* (Moonlight et al. 2020), there still exists obscurity between lineages. Phylogenetic analysis based on the chloroplast genome is urgently required to offer the potential to shed light on the evolution of this species-rich group. *Begonia coptidifolia* Ye et al. (2004) is a newly reported species found in Southern China (Ye et al. 2004). It is a small herb, 20–30 cm tall, generally discovered to grow in moisture-rich environments, such as along streams. Up to now, no phylogenetic studies have been carried out on this species. Therefore, we report here the complete chloroplast genomic sequence of *B. coptidifolia* to provide a resource for future better resolution of its relationship with the other *Begonia* species.

Leaf samples of *B*. *coptidifolia* were collected from Ehuangzhang, Yangchun City, China (21°50′36″N, 111°21′29″E), and a specimen was stored at the Herbarium of South China Botanical Garden (No IBSC-T-20200317015). Using a 2 × 150 bp paired-end sequencing strategy, about 17 Gb whole- genome sequencing reads were produced from this sample on the Illumina HiSeq X Ten (Illumina, San Diego, CA, USA) platform. With NOVOPlasty 4.2.1 (Dierckxsens et al. 2017), the chloroplast genome of *B*. *coptidifolia* was then assembled based on these reads. After assembly, the genome was annotated by CPGAVAS2 (Shi et al. 2019). The assembled chloroplast genome and its detailed annotations were submitted to GenBank under the accession number MW080659. A phylogenetic tree for *B*. *coptidifolia* and another 26 species (Figure 1) in Cucurbitales was generated by mashtree v1.2.0 (Katz et al. 2019), which can perform rapid genome sequences comparison by calculating their pairwise distances with Mash (Ondov et al. 2016) and then construct a dendrogram using neighbor-joining (NJ) algorithm (e.g. Guo et al. 2020).

**Figure 1.**
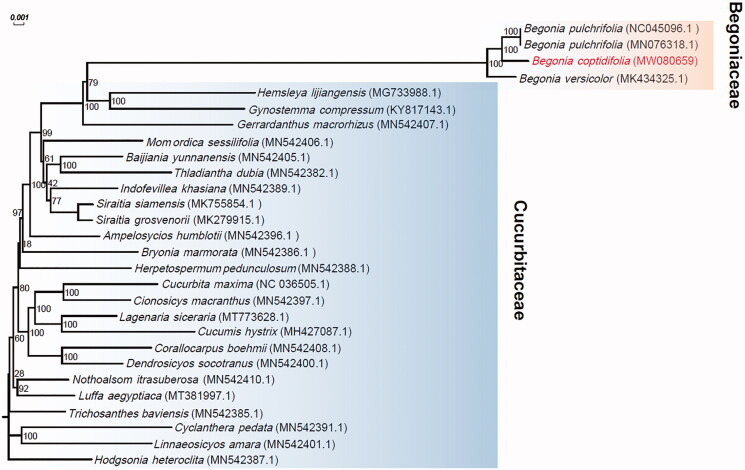
Phylogenetic analysis based on the complete chloroplast genome sequences of *Begonia coptidifolia* and 26 additional species in Cucurbitales with their GenBank accession numbers denoted in parentheses. Bootstrap percentages (1000 replicates) are shown at nodes.

The assembled chloroplast of *B*. *coptidifolia* is a closed circular molecule with a length of 169,412bp. The overall GC content is 35.57%. The genome contains a large single-copy region of 75,937 bp, a small single-copy region of 18,362 bp, and two inverted repeat regions (IRA and IRB) of 37,556 bp and 37,557 bp, respectively. Annotation by CPGAVAS2 identified a total of 130 genes, including 82 protein-coding genes, 8 ribosomal RNA genes, and 40 transfer RNA genes. Phylogenetic analysis revealed that *Begonia* forms a monophyletic group close to *Hemsleya lijiangensis* and *Gynostemma compressum*, and *B*. *coptidifolia* is a sister species to *B*. *pulchrifolia* (Figure 1).

## Data Availability

The complete chloroplast genome sequences of *Begonia coptidifolia* have been deposited in GenBank under the accession number MW080659 and is also accessible at https://doi.org/10.13140/RG.2.2.36088.47361. The associated BioProject, SRA, and Bio-Sample numbers are PRJNA682519, SRR13197233, and SAMN16992810 respectively.

## References

[CIT0001] Dierckxsens N, Mardulyn P, Smits G. 2017. NOVOPlasty: de novo assembly of organelle genomes from whole genome data. Nucleic Acids Res. 45(4):e18. 28204566 10.1093/nar/gkw955 PMC5389512

[CIT0002] Guo Y, Wang Z-F, Cao H-L. 2020. The complete chloroplast genome sequence of *Ormosia boluoensis*. Mitochondrial DNA B. 5(1):999–1000. 10.1080/23802359.2020.1720541 PMC7748850 33366845

[CIT0003] Katz LS, Griswold T, Morrison SS, Caravas JA, Zhang S, den Bakker HC, Deng X, Carleton HA. 2019. Mashtree: a rapid comparison of whole genome sequence files. JOSS. 4(44):1762. 10.21105/joss.01762 PMC9380445 35978566

[CIT0004] Moonlight PW, Holland R, Cano A, Purvis DA. 2020. A new species of tuberous *Begonia* (Begoniaceae) from Andean Peru. Edinb J Bot. 77(1):145–159.

[CIT0005] Ondov BD, Treangen TJ, Melsted P, Mallonee AB, Bergman NH, Koren S, Phillippy AM. 2016. Mash: fast genome and metagenome distance estimation using MinHash. Genome Biol. 17(1):132. 27323842 10.1186/s13059-016-0997-x PMC4915045

[CIT0006] Shi L, Chen H, Jiang M, Wang L, Wu X, Huang L, Liu C. 2019. CPGAVAS2, an integrated plastome sequence annotator and analyzer. Nucleic Acids Res. 47(W1):W65–W73. 31066451 10.1093/nar/gkz345 PMC6602467

[CIT0007] Ye H-G, Wang F-G, Ye Y-S, Peng C-I. 2004. *Begonia coptidifolia*, a new species from China. Bot Bull Acad Sinica. 45:259–266.

